# Multisensor Super Resolution Using Directionally-Adaptive Regularization for UAV Images

**DOI:** 10.3390/s150512053

**Published:** 2015-05-22

**Authors:** Wonseok Kang, Soohwan Yu, Seungyong Ko, Joonki Paik

**Affiliations:** Department of Image, Chung-Ang University, 84 Heukseok-ro, Dongjak-gu, Seoul 156-756, Korea; E-Mails: kandws12@cau.ac.kr (W.K.); shyu@cau.ac.kr (S.Y.); fantaaltanix@cau.ac.kr (S.K.)

**Keywords:** multisensor super-resolution (SR), UAV image enhancement, regularized image restoration, image fusion

## Abstract

In various unmanned aerial vehicle (UAV) imaging applications, the multisensor super-resolution (SR) technique has become a chronic problem and attracted increasing attention. Multisensor SR algorithms utilize multispectral low-resolution (LR) images to make a higher resolution (HR) image to improve the performance of the UAV imaging system. The primary objective of the paper is to develop a multisensor SR method based on the existing multispectral imaging framework instead of using additional sensors. In order to restore image details without noise amplification or unnatural post-processing artifacts, this paper presents an improved regularized SR algorithm by combining the directionally-adaptive constraints and multiscale non-local means (NLM) filter. As a result, the proposed method can overcome the physical limitation of multispectral sensors by estimating the color HR image from a set of multispectral LR images using intensity-hue-saturation (IHS) image fusion. Experimental results show that the proposed method provides better SR results than existing state-of-the-art SR methods in the sense of objective measures.

## 1. Introduction

Multispectral images contain complete spectrum information at every pixel in the image plane and are currently applied to various unmanned aerial vehicle (UAV) imaging applications, such as environmental monitoring, weather forecasting, military intelligence, target tracking, *etc*. However, it is not easy to acquire a high-resolution (HR) image using a multispectral sensor, because of the physical limitation of the sensor. A simple way to enhance the spectral resolution of a multispectral image is to increase the number of photo-detectors at the cost of sensitivity and signal-to-noise ratio due to the reduced size of pixels. In order to overcome such physical limitations of a multispectral imaging sensor, an image fusion-based resolution enhancement method is needed [1,2].

Various enlargement and super-resolution (SR) methods have been developed in many application areas over the past few decades. The goal of these methods is to estimate an HR image from one or more low-resolution (LR) images. They can be classified into two groups: (i) single image based; and (ii) multiple image based. The latter requires a set of LR images to reconstruct an HR image. It performs the warping process to align multiple LR images with a sub-pixel precision. If LR images are degraded by motion blur and additive noise, the registration process becomes more difficult. To solve this problem, single image-based SR methods became popular, including: an interpolation-based SR [3–6], patch-based SR [7–12], image fusion-based SR [13–17], and others [18].

In order to solve the problem of simple interpolation-based methods, such as linear and cubic-spline interpolation [3], a number of improved and/or modified versions of image interpolation methods have been proposed in the literature. Li *et al.* used the geometric duality between the LR and HR images using local variance in the LR image [4]. Zhang *et al.* proposed an edge-guided non-linear interpolation algorithm using directionally-adaptive filters and data fusion [5]. Giachetti *et al.* proposed a curvature-based iterative interpolation using a two-step grip filling and an iterative correction of the estimated pixels [6]. Although the modified versions of interpolation methods can improve the image quality in the sense of enhancing the edge sharpness and visual improvement to a certain degree, fundamental interpolation artifacts, such as blurring and jagging, cannot be completely removed, due to the nature of the interpolation framework.

Patch-based SR methods estimate an HR image from the LR image, which is considered as a noisy, blurred and down-sampled version of the HR image. Freeman *et al.* proposed the example-based SR algorithm using the hidden Markov model that estimates the optimal HR patch corresponding to the input LR patch from the external training dataset [7]. Glasner *et al.* used a unified SR framework using patch similarity between in- and cross-scale images in the scale space [8]. Yang *et al.* used patch similarity from the learning dataset of HR and LR patch pairs in the sparse representation model [9]. Kim *et al.* proposed a sparse kernel regression-based SR method using kernel matching pursuit and gradient descent optimization to map the pairs of trained example patches from the input LR image to the output HR image [10]. Freedman *et al.* used non-dyadic filter banks to preserve the property of an input LR image and searched a similar patch using local self-similarity in the locally-limited region [11]. He *et al.* proposed a Gaussian regression-based SR method using soft clustering based on the local structure of pixels [12]. Existing patch-based SR methods can better reduce the blurring and jagging artifacts than interpolation-based SR methods. However, non-optimal patches make the restored image look unnatural, because of the inaccurate estimation of the high-frequency components.

On the other hand, image fusion-based SR methods have been proposed in the remote sensing fields. The goal of these methods is to improve the spatial resolution of LR multispectral images using the detail of the corresponding HR panchromatic image. Principal component analysis (PCA)-based methods used the projection of the image into the differently-transformed space [13]. Intensity-hue-saturation (IHS) [14,15] and Brovery [17] methods considered the HR panchromatic image as a linear combination of the LR multispectral images. Ballester *et al.* proposed an improved variational-based method [16]. These methods assume that an HR panchromatic image is a linear combination of LR multispectral images. Therefore, conventional image fusion-based SR methods have the problem of using an additional HR panchromatic imaging sensor.

In order to improve the performance of the fusion-based SR methods, this paper presents a directionally-adaptive regularized SR algorithm. Assuming that the HR monochromatic image is a linear combination of multispectral images, the proposed method consists of three steps: (i) acquisition of monochromatic LR images from the set of multispectral images; (ii) restoration of the monochromatic HR image using the proposed directionally-adaptive regularization; and (iii) reconstruction of the color HR image using image fusion. The proposed SR algorithm is an extended version of the regularized restoration algorithms proposed in [19,20] for optimal adaptation to directional edges and uses interpolation algorithms in [21–23] for resizing the interim images at each iteration.

The major contribution of this work is two-fold: (i) the proposed method can estimate the monochromatic HR image using directionally-adaptive regularization that provides the optimal adaptation to directional edges in the image; and (ii) it uses an improved version of the image fusion method proposed in [14,15] to reconstruct the color HR image. Therefore, the proposed method can generate a color HR image without additional high-cost imaging sensors using image fusion and the proposed regularization-based SR method. In experimental results, the proposed SR method is compared with seven existing image enlargement methods, including interpolation-based, example-based SR and patch similarity-based SR methods in the sense of objective assessments.

The rest of this paper is organized as follows. Section 2 summarizes the theoretical background of regularized image restoration and image fusion. Section 3 presents the proposed directional adaptive regularized SR algorithm and image fusion. Section 4 summarizes experimental results on multi- and hyper-spectral images, and Section 5 concludes the paper.

## 2. Theoretical Background

The proposed multispectral SR framework is based on regularized image restoration and multispectral image fusion. This section presents the theoretical background of multispectral image representation, regularized image restoration and image fusion in the following subsection.

### 2.1. Multispectral Image Representation

A multispectral imaging sensor measures the radiance of multiple spectral bands whose range is divided into a series of contiguous and narrow spectral bands. On the other hand, a monochromatic or single-band imaging sensor measures the radiance of the entire spectrum of the wavelength. The relationship between multispectral and monochromatic images is assumed to be modeled as the gray-level images between wavelength *ω*_1_ and *ω*_2_ as [24]:

(1) $$I=\int_{\omega_{1}}^{\omega_{2}}R\left(\omega \right)Kq\left(\omega \right)d\omega +\eta_{\left(\omega_{1}∼\omega_{2}\right)}$$

where *R*(*ω*) represents the spectral radiance through the sensor's entrance pupil and *K* a constant that is determined by the sensor characteristics, including the electronic gain, detector saturation, quantization levels and the area of the aperture. *q*(*ω*) is the spectral response function of the sensor in the wavelength range between *ω*_1_ and *ω*_2_*. η*_(*ω*1∼*ω*2)_ is the noise generated by the dark signal.

**Figure 1:**
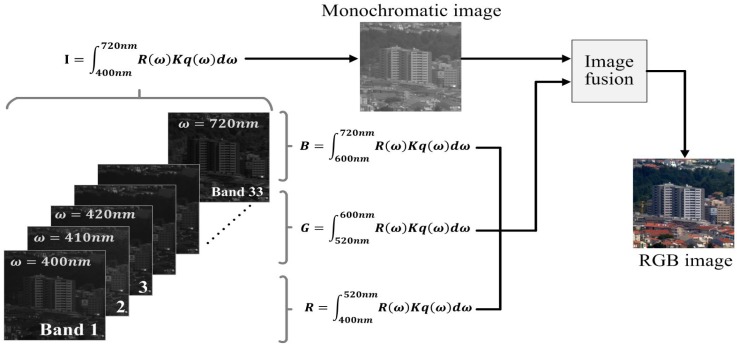
The multispectral imaging process.

Since the spectral radiance *R*(*ω*) does not change by the sensor, the initial monochromatic HR image is generated by Equation (1), and it is used to reconstruct the monochromatic HR image from multispectral LR images using image fusion [14,15]. Figure 1 shows the multispectral imaging process, where a panchromatic image is acquired by integrating the entire spectral band, and the corresponding RGB image is also acquired using, for example, 33 bands. The high-resolution (HR) panchromatic and low-resolution (LR) RGB image are fused to generate an HR color image.

### 2.2. Multispectral Image Fusion

In order to improve the spatial resolution of multispectral images, the intensity-hue-saturation (IHS) image fusion method is widely used in remote sensing fields [14,15]. More specifically, this method converts a color image into the IHS color space, where only the intensity band is replaced by the monochromatic HR image. The resulting HR image is obtained by converting the replaced intensity and the original hue and saturation back to the RGB color space.

### 2.3. Regularized Image Restoration

Regularization-based image restoration or enlargement algorithms regard the noisy, LR images as the output of a general image degradation process and incorporate *a priori* constraints into the restoration process to make the inverse problem better posed [19–23].

The image degradation model for a single LR image can be expressed as:

(2) g=Hf+η

where *g* represents the observed LR image, *H* the combined low-pass filtering and down-sampling operator, *f* the original HR image and *η* the noise term.

The restoration problem is to estimate the HR image *f* from the observed LR image *g*. Therefore, the regularization approach minimizes the cost function as:

(3) $$\begin{array}{ll} J\left(f\right) & =\frac{1}{2}\left\{{\left\|g-Hf\right\|}^{2}\right\}+\frac{1}{2}\lambda {\left\|Cf\right\|}^{2}\\ & =\frac{1}{2}{\left(g-Hf\right)}^{T}\left(g-Hf\right)+\frac{1}{2}\lambda f^{T}C^{T}Cf \end{array}$$

where *C* represents a two-dimensional (2D) high-pass filter, λ is the regularization parameter and ‖*Cf*‖^2^ the energy of the high-pass filtered image representing the amount of noise amplification in the restoration process.

The derivative of Equation (3) with respect to *f* is computed as:

(4) $$\nabla J\left(f\right)=\left(-H^{T}g+H^{T}Hf\right)+\lambda C^{T}Cf$$

which becomes zero if:

(5) $$f={\left(H^{T}H+\lambda C^{T}C\right)}^{-1}H^{T}g$$

Thus, Equation (5) can be solved using the well-known regularized iteration process as:

(6) $$f^{k+1}=f^{k}+\beta \left\{H^{T}g-\left(H^{T}H+\lambda C^{T}C\right)f^{k}\right\}$$

where *H^T^H* + λ*C^T^C* represents the better-posed system matrix, and the step length *β* should be sufficiently small for the convergence.

## 3. Directionally-Adaptive Regularization-Based Super-Resolution with Multiscale Non-Local Means Filter

Since the estimation of the original HR image from the image degradation process given in (2) is almost always an ill-posed problem, there is no unique solution, and a simple inversion process, such as inverse filtering, results in significant amplification of noise and numerical errors [7–12]. To solve this problem, regularized image restoration incorporates *a priori* constraints on the original image to make the inverse problem better posed.

**Figure 2:**
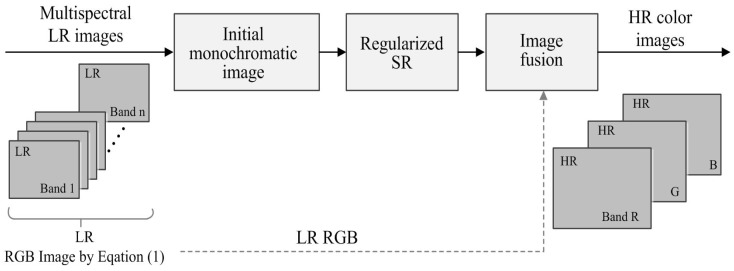
Block-diagram of the proposed super-resolution method.

In this section, we describe a modified version of the regularized SR algorithm using a non-local means (NLM) filter [25] and the directionally-adaptive constraint as a regularization term to preserve edge sharpness and to suppress noise amplification. The reconstructed monochromatic HR image is used to generate a color HR image together with given LR multispectral images using IHS image fusion [14,15]. The block-diagram of the proposed method is shown in Figure 2.

### 3.1. Multispectral Low-Resolution Image Degradation Model

Assuming that the original monochromatic image is a linear combination of multispectral images [24], the observed LR multispectral images are generated by low-pass filtering and down-sampling from the differently translated version of the original HR monochromatic image. More specifically, the observed LR image in the *i*-th multispectral band is defined as:

(7) $$\begin{array}{cc} g_{i}=Hf_{\left(x_{i},y_{i}\right)}^{\prime }+\eta_{i}=H_{i}f+\eta ,\;\text{for}\;, & i=1,\ldots ,L \end{array}$$

where $f_{\left(x_{i},y_{i}\right)}^{\prime }$ represents the translated version of the original HR monochromatic image *f* by (*x_i_*, *y_i_*), *H* the image degradation operator, including both low-pass filtering and down-sampling, and *η* the additive noise. In this paper, we assume that there is no warping operation in the image degradation model, because LR images are acquired by the multispectral sensor for the same scene.

### 3.2. Multiscale Non-Local Means Filter

If noise is present in the image degradation model given in Equation (2), the estimated HR image using the simple inverse filter yields:

(8) $$\hat{f}=H^{-1}g=H^{-1}\left(Hf-\eta \right)=f^{*}+\Delta f$$

where the term Δ*f* = *H*^−1^*η* amplifies the noise in an uncontrollable manner. This process can be considered to solve *g* = *Hf* with the observation noise or perturbation *η*, which result in the amplified error Δf = *H*^−1^*η* in the solution.

If Δ*f* is unbounded, the corresponding image restoration of the SR problem is ill posed. To solve the ill-posed restoration problem, we present an improved multiscale non-local means (NLM) filter to minimize the noise before the main restoration problem. The estimated noise-removed monochromatic image can be obtained using the least-squares optimization as:

(9) $$\begin{array}{cc} {\hat{f}}_{m}=\arg\underset{f_{m}}{\min}\underset{n\in \Omega_{s}}{\text{∑}}\left[g_{s,n}^{\text{P}}-f_{m}\right]w_{m,n}^{\text{P}}, & \text{for},\;m,n=1,\ldots ,M,N \end{array}$$

where *f_m_* represents the *m*-th underlying pixel, Ω*_s_* the local region in the 1.25*_S_*-times down-scaled image, for *s* = {−1,−2,−3}, using the cubic-spline interpolation kernel [3], $g_{s,n}^{\text{P}}$ the local patches of *g_m_* corresponding to Ω*_s_*, $w_{m,n}^{\text{P}}$ the similarity weighting value between the local patches $g_{s,n}^{\text{P}}$ in the down-scaled image and the corresponding patch in *g_m_* and the superscript **P** a patch.

Since the cubic-spline kernel performs low-pass filtering, it decreases the noise variance and guarantees searching sufficiently similar patches [8]. The similarity weight value is computed in the down-scaled image as:

(10) $$w_{m,n}^{\text{P}}=\exp\left(-\frac{{\left\|g_{s,n}^{\text{P}}-g_{m}^{\text{P}}\right\|}_{\text{G}}^{2}}{1.25^{s}\sigma^{2}}\right)$$

where $g_{s,n}^{\text{P}}$ represents pixels in the patch centered at the location of *g_s,m_* and $g_{m}^{\text{P}}$ pixel in the patch centered at the location of *g_m_* in the original scale image. The parameter **G** is a Gaussian kernel that controls the exponential decay in the weighting computation.

The solution of the least-squares estimation in Equation (9) is given as:

(11) $${\hat{f}}_{m}={\left(\underset{n\in \Omega_{s}}{\text{∑}}w_{m,n}^{\text{P}}\right)}^{-1}\left(\underset{n\in \Omega_{s}}{\text{∑}}w_{m,n}^{\text{P}}g_{s,n}^{\text{P}}\right)$$

### 3.3. Directionally-Adaptive Constraints

In minimizing the cost function in Equation (3), minimization of ‖*g* − *Hf*‖^2^ results in noise amplification, while minimization of ‖*Cf*‖**^2^** results in a non-edge region. In this context, conventional regularized image restoration or SR algorithms [21–23] tried to estimate the original image by minimizing the cost function that is a linear combination of the two energies as ‖*g* − *Hf*‖^2^ + λ‖*Cf*‖^2^. In this paper, we incorporate directionally-adaptive smoothness constraints into regularization process to preserve directional edge sharpness and to suppress noise amplification as:

(12) $$\begin{array}{cc} \lambda {\left\|C_{D}f\right\|}^{2}, & \text{for}\;D=1,\ldots ,5 \end{array}$$

where the directionally-adaptive constraints *C_D_*, for *D* = 1,…, 5, suppress the noise amplification along the corresponding edge direction. In this work, we use the edge orientation classification filter [23]. The proposed directionally-adaptive constraints can be implemented using four 2D different high-pass filters as:

(13) $$C_{1}^{0^{{}^{\circ}}}=\left(\begin{array}{ccc} 0 & 0 & 0\\ 0 & 1 & 0\\ 0 & 0 & 0 \end{array}\right)-\frac{1}{6}\left(\begin{array}{ccc} 0 & 0 & 0\\ 1 & 1 & 1\\ 1 & 1 & 1 \end{array}\right)=\left(\begin{array}{ccc} 0 & 0 & 0\\ -0.1677 & 0.8333 & -0.1677\\ -0.1677 & 0.8333 & -0.1677 \end{array}\right)$$

(14) $$C_{2}^{45^{{}^{\circ}}}=\left(\begin{array}{ccc} 0 & 0 & 0\\ 0 & 1 & 0\\ 0 & 0 & 0 \end{array}\right)-\frac{1}{6}\left(\begin{array}{ccc} 1 & 0 & 0\\ 1 & 1 & 1\\ 1 & 1 & 1 \end{array}\right)=\left(\begin{array}{ccc} -0.1677 & 0 & 0\\ -0.1677 & 0.8333 & 0\\ -0.1677 & 0.8333 & -0.1677 \end{array}\right)$$

(15) $$C_{3}^{90^{{}^{\circ}}}=\left(\begin{array}{ccc} 0 & 0 & 0\\ 0 & 1 & 0\\ 0 & 0 & 0 \end{array}\right)-\frac{1}{6}\left(\begin{array}{ccc} 0 & 1 & 1\\ 0 & 1 & 1\\ 0 & 1 & 1 \end{array}\right)=\left(\begin{array}{ccc} 0 & -0.1677 & -0.1677\\ 0 & 0.8333 & -0.1677\\ 0 & -0.1677 & -0.1677 \end{array}\right)$$

and:

(16) $$C_{4}^{135^{{}^{\circ}}}=\left(\begin{array}{ccc} 0 & 0 & 0\\ 0 & 1 & 0\\ 0 & 0 & 0 \end{array}\right)-\frac{1}{6}\left(\begin{array}{ccc} 1 & 1 & 1\\ 0 & 1 & 1\\ 0 & 1 & 1 \end{array}\right)=\left(\begin{array}{ccc} -0.1677 & -0.1677 & -0.1677\\ 0 & 0.8333 & -0.1677\\ 0 & 0 & -0.1677 \end{array}\right)$$

By applying the directionally-adaptive constraints, an HR image can be restored from the input LR image. In the restored HR image, four directional edges are well preserved. In order to suppress noise amplification in the non-edge (NE) regions, the following constraint is used.

(17) $$C_{5}^{\text{NE}}=\left(\begin{array}{ccc} 0 & 0 & 0\\ 0 & 1 & 0\\ 0 & 0 & 0 \end{array}\right)-\frac{1}{9}\left(\begin{array}{ccc} 1 & 1 & 1\\ 1 & 1 & 1\\ 1 & 1 & 1 \end{array}\right)=\left(\begin{array}{ccc} -0.1111 & -0.1111 & -0.1111\\ -0.1111 & 0.8333 & -0.1111\\ -0.1111 & -0.1111 & -0.1111 \end{array}\right)$$

### 3.4. Combined Directionally-Adaptive Regularization and Modified Non-Local Means Filter

Given the multispectral LR images *g_j_*, for *i* = 1,…, *L*, the estimated monochromatic HR image *f̂* is given by the following optimization:

(18) $$\hat{f}=\arg\underset{f}{\min}J\left(f\right)$$

where the multispectral extended version of Equation (3) is given as:

(19) $$\begin{array}{ll} J\left(f\right) & =\frac{1}{2}\overset{L}{\underset{i=1}{\text{∑}}}{\left\|g_{i}-H_{i}f\right\|}^{2}+\frac{\lambda }{2}{\left\|C_{D}f\right\|}^{2}\\ & =\frac{1}{2}\overset{L}{\underset{i=1}{\text{∑}}}{\left(g_{i}-H_{i}f\right)}^{T}\left(g_{i}-H_{i}f\right)+\frac{\lambda }{2}f^{T}C_{D}^{T}C_{D}f \end{array}$$

The derivative of Equation (19) with respect to *f* is computed as:

(20) $$\begin{array}{ll} \nabla J\left(f\right) & =\overset{L}{\underset{i=1}{\text{∑}}}\left(-H_{i}^{T}g_{i}+H_{i}^{T}H_{i}f\right)+\lambda C_{D}^{T}C_{D}f\\ & =\left\{\overset{L}{\underset{i=1}{\text{∑}}}H_{i}^{T}H_{i}+\lambda C_{D}^{T}C_{D}\right\}f-\overset{L}{\underset{i=1}{\text{∑}}}H_{i}^{T}g_{i} \end{array}$$

which becomes zero if:

(21) $$f={\left(\overset{L}{\underset{i=1}{\text{∑}}}H_{i}^{T}H_{i}+\lambda C_{D}^{T}C_{D}\right)}^{-1}\overset{L}{\underset{i=1}{\text{∑}}}H_{i}^{T}g_{i}$$

Finally, Equation (21) can be solved using the well-known iterative optimization with the proposed multiscale NLM filter as:

(22) $$f^{k+1}=f_{\text{NLM}}^{k}+\beta \left\{\overset{L}{\underset{i=1}{\text{∑}}}H_{i}^{T}g_{i}-\left(\overset{L}{\underset{i=1}{\text{∑}}}H_{i}^{T}H_{i}+\lambda C_{D}^{T}C_{D}\right)f_{\text{NLM}}^{k}\right\}$$

where the matrix ${\text{∑}}_{i=1}^{L}H_{i}^{T}H_{i}+\lambda C_{D}^{T}C_{D}$ is better-conditioned, and the step length *β* should be small enough to guarantee the convergence. $f_{\text{NLM}}^{k}$ represents the multiscale NLM filtered version that can be expressed as:

(23) $$f_{\text{NLM}}^{k}=\overset{M}{\underset{m=1}{\text{∑}}}\left[{\left(\underset{n\in \Omega_{s}}{\text{∑}}w_{m,n}^{\text{P}}\right)}^{-1}\left\{\underset{n\in \Omega_{s}}{\text{∑}}w_{m,n}^{\text{P}}\left(\overset{L}{\underset{i=1}{\text{∑}}}H_{i}^{T}g_{s,i,n}^{\text{P}}\right)\right\}\right]$$

For the implementation of Equation (22), the term $H_{i}^{T}g_{i}=S_{i}^{T}H^{T}g_{i}$ implies that the *i*-th multispectral LR images are first enlarged by simple interpolation as:

(24) $$H^{T}={\tilde{H}}^{T}⊗{\tilde{H}}^{T}$$

where ⨂ represents the Kronecker product of matrices and *H̃* represents the one-dimensional (1D) low-pass filtering and subsampling process with a specific magnification ratio.

In order to represent the geometric misalignment among different spectral bands, pixel shifting by (−*x_i_*, −*y_i_*) is expressed as *S_i_* = *S̃_xi_* ⨂ *S̃_yi_*, where *S̃_p_* is the 1D translating matrix that shifts a 1D vector by *p* samples. The term $H_{i}^{T}H_{i}f^{k}=S_{i}^{T}H^{T}HS_{i}f^{k}$ implies that the *k*-th iterative solution is shifted by (*x_i_*, *y_i_*), down-sampled by *H*, enlarged by interpolation *H^T^* and then shifted by (−*x_i_*, −*y_i_*), respectively.

### 3.5. Image Fusion-Based HR Color Image Reconstruction

A multispectral imaging sensor measures the radiance of multiple spectral bands whose ranges are divided into a series of contiguous and narrow spectral bands. In this paper, we adopt the IHS fusion method mentioned to estimate the HR color image from multispectral LR images as [14,15]:

(25) $$\left[\begin{array}{c} \text{I}\\ \text{H}\\ \text{S} \end{array}\right]=\left[\begin{array}{ccc} \frac{1}{3} & \frac{1}{3} & \frac{1}{3}\\ \frac{-\sqrt{2}}{6} & \frac{-\sqrt{2}}{6} & \frac{2\sqrt{2}}{6}\\ \frac{-1}{\sqrt{2}} & \frac{-1}{\sqrt{2}} & 0 \end{array}\right]\;\left[\begin{array}{c} \text{R}\\ \text{G}\\ \text{B} \end{array}\right]$$

where R, G and B bands are computed as:

(26) $$R=\int_{400\text{nm}}^{520\text{nm}}R\left(\omega \right)Kq\left(\omega \right)d\omega$$

(27) $$G=\int_{520\text{nm}}^{600\text{nm}}R\left(\omega \right)Kq\left(\omega \right)d\omega$$

and:

(28) $$B=\int_{600\text{nm}}^{720\text{nm}}R\left(\omega \right)Kq\left(\omega \right)d\omega$$

where *R*(*ω*) represents the spectral radiance, *K* a constant gain and *q*(*ω*) is the spectral response function of the multispectral sensor as defined in Equation (1). The intensity component I is replaced with the estimated monochromatic HR image, and then, the IHS color space is converted back to the RGB color space as:

(29) $$\left[\begin{array}{c} R_{H}\\ G_{H}\\ B_{H} \end{array}\right]=\left[\begin{array}{ccc} 1 & \frac{-1}{\sqrt{2}} & \frac{1}{\sqrt{2}}\\ 1 & \frac{-1}{\sqrt{2}} & \frac{-1}{\sqrt{2}}\\ 1 & \sqrt{2} & 0 \end{array}\right]\;\left[\begin{array}{c} \hat{f}\\ \text{H}\\ \text{S} \end{array}\right]$$

where *f̂* represents the estimated monochromatic HR image and *C_H_*, for *C* ∈ {*R, G, B*}, is the fused color HR image.

**Figure 3:**
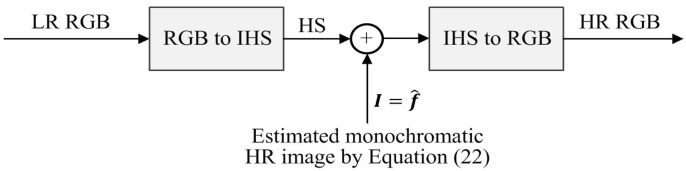
Block-diagram of the proposed fusion-based high-resolution (HR) color image reconstruction process.

In this paper, we used the cubic-spline interpolation method to enlarge the hue (H) and saturation (S) channels by the given magnification factor. Figure 3 shows the image fusion-based HR color image reconstruction process.

## 4. Experimental Results

In this section, the proposed method is tested on various simulated, multispectral, real UAV and remote sensing images to evaluate the SR performance. In the following experiments, parameters were selected to produce the visually best results. In order to provide comparative experimental results, various existing interpolation and state-of-the-art SR methods were tested, such as cubic-spline interpolation [3], advanced interpolation-based SR [4–6], example-based SR [7] and patch-based SR [9–12].

To compare the performance of several SR methods, we used a set of full-reference metrics of image quality, including peak-to-peak signal-to-noise ratio (PSNR), structural similarity index measure (SSIM) [26], multiscale-SSIM (MS-SSIM) [27] and feature similarity index (FSIM) [28]. On the other hand, for the evaluation of the magnified image quality without the reference HR image, we adopted the completely blind image quality assessment methods, including the blind/referenceless image spatial quality evaluator (BRISQUE) [29] and natural image quality evaluator (NIQE) [30]. The higher image quality results in lower BRISQUE and NIQE values, but higher PSNR, SSIM, MS-SSIM and FSIM values.

The BRISQUE quantifies the amount of naturalness using the locally-normalized luminance values on *a priori* knowledge of both natural and artificially-distorted images. The NIQE takes into account the amount of deviations from the statistical regularities observed in the undistorted natural image contents using statistical features in natural scenes. Since BRISQUE and NIQE are referenceless metrics, they may not give the same ranking to the well-known metrics with reference, such as PSNR and SSIM.

### 4.1. Experiment Using Simulated LR Images

**Figure 4:**
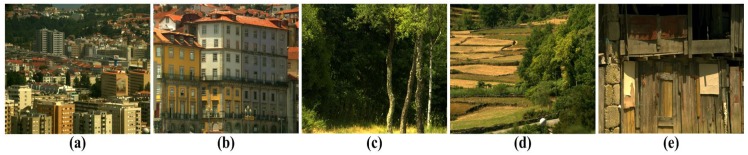
Five multispectral test images.

In order to evaluate the qualitative performance of various SR algorithms, we used five multispectral test images, each of which consists of 33 spectral bands in the wavelength range from 400 to 720 nanometers (nm), as shown Figure 4. In order to compare the objective image quality measures, such as PSNR, SSIM, MS-SSIM, FSIM and NIQE, the original multispectral HR image is first down-sampled by a factor of four to simulate the input LR images. Next, the input LR images are magnified four times using the nine existing methods and the proposed multispectral SR method.

In simulating LR images, the discrete approximation of Equation (1) is used, and the RGB color image is degraded by Equation (2). Given the simulated LR images, existing SR algorithms enlarge the RGB channels, whereas the proposed method generates the monochromatic HR image, including all spectral wavelengths, using the directionally-adaptive SR algorithm, and the IHS image fusion finally generates the color HR image using the monochromatic HR and RGB LR images [14,15].

**Figure 5:**
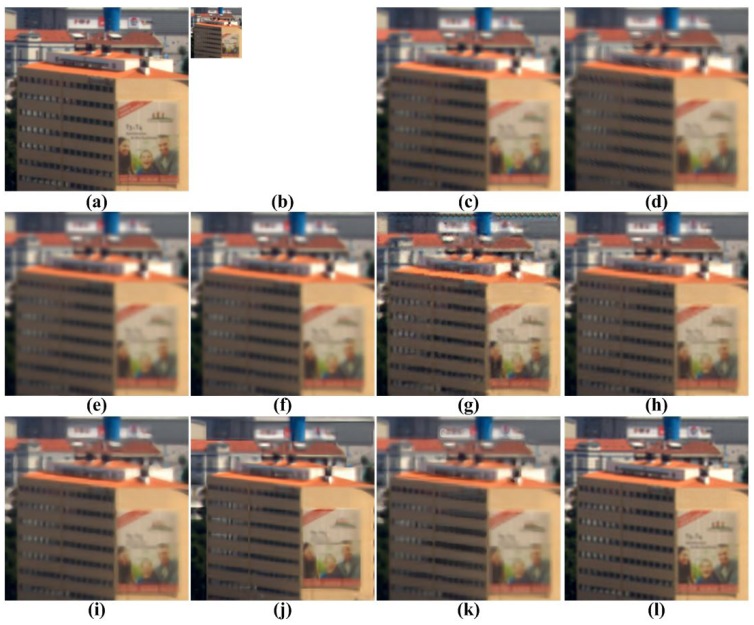
Results of resolution enhancement by enlarging a simulated low-resolution (LR) multispectral image: (**a**) cropped original HR image in Figure 4a; (**b**) the four-times down-sampled LR image; results of: (**c**) cubic-spline interpolation [3]; (**d**) interpolation-based SR [4]; (**e**) interpolation-based SR [5]; (**f**) interpolation-based SR [6]; (**g**) example-based SR [7]; (**h**) patch-based SR [9]; (**i**) patch-based SR [10]; (**j**) patch-based SR [11]; (**k**) patch-based SR [12] and (**l**) the proposed method.

**Figure 6:**
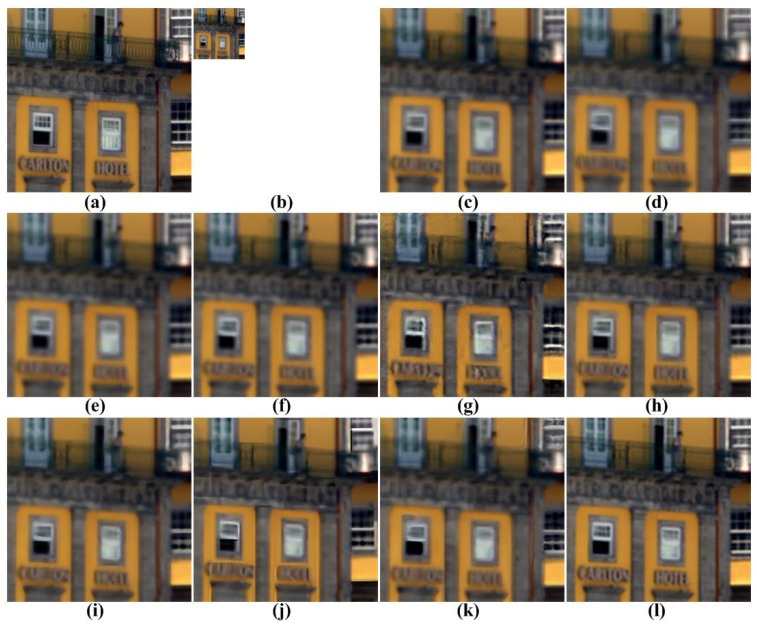
Results of resolution enhancement by enlarging a simulated LR multispectral image: (**a**) cropped original HR image in Figure 4b; (**b**) the four-times down-sampled LR image; results of: (**c**) cubic-spline interpolation [3], (**d**) interpolation-based SR [4], (**e**) interpolation-based SR [5], (**f**) interpolation-based SR [6], (**g**) example-based SR [7], (**h**) patch-based SR [9], (**i**) patch-based SR [10], (**j**) patch-based SR [11], (**k**) patch-based SR [12] and (**l**) the proposed method.

**Figure 7:**
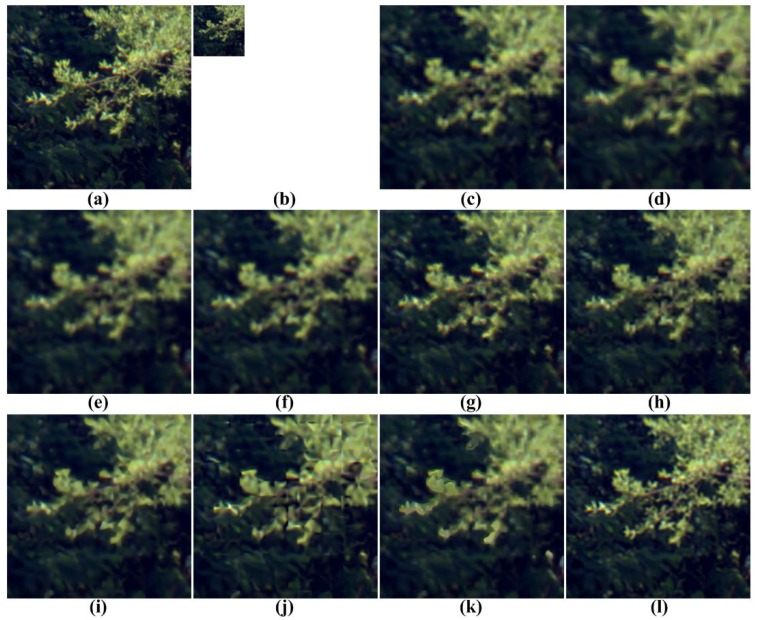
Results of resolution enhancement by enlarging a simulated LR multispectral image: (**a**) cropped original HR image in Figure 4c; (**b**) the four-times down-sampled LR image; results of: (c) cubic-spline interpolation [3], (**d**) interpolation-based SR [4], (**e**) interpolation-based SR [5], (**f**) interpolation-based SR [6], (**g**) example-based SR [7], (**h**) patch-based SR [9], (**i**) patch-based SR [10], (**j**) patch-based SR [11], (**k**) patch-based SR [12] and (**l**) the proposed method.

**Figure 8:**
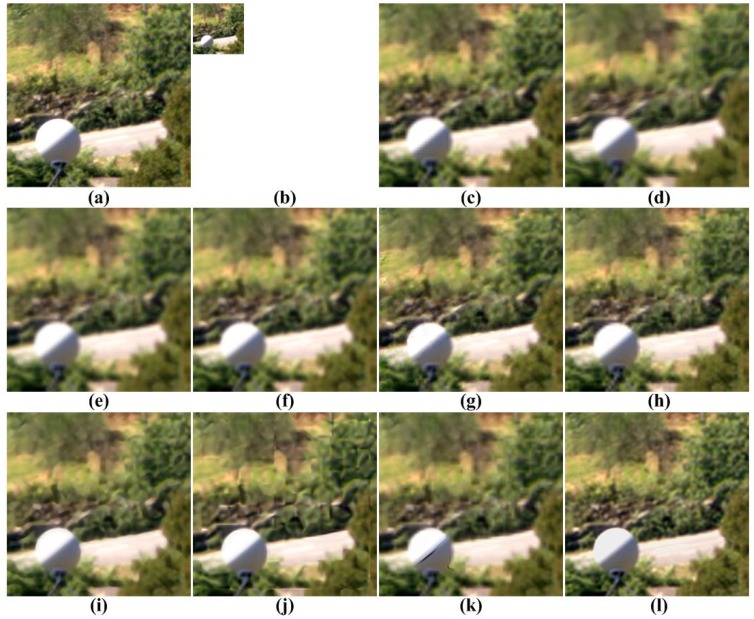
Results of resolution enhancement by enlarging a simulated LR multispectral image: (**a**) cropped original HR image in Figure 4d; (**b**) the four-times down-sampled LR image; results of: (**c**) cubic-spline interpolation [3], (**d**) interpolation-based SR [4], (**e**) interpolation-based SR [5], (**f**) interpolation-based SR [6], (**g**) example-based SR [7], (**h**) patch-based SR [9], (**i**) patch-based SR [10], (**j**) patch-based SR [11], (**k**) patch-based SR [12] and (**l**) the proposed method.

**Figure 9:**
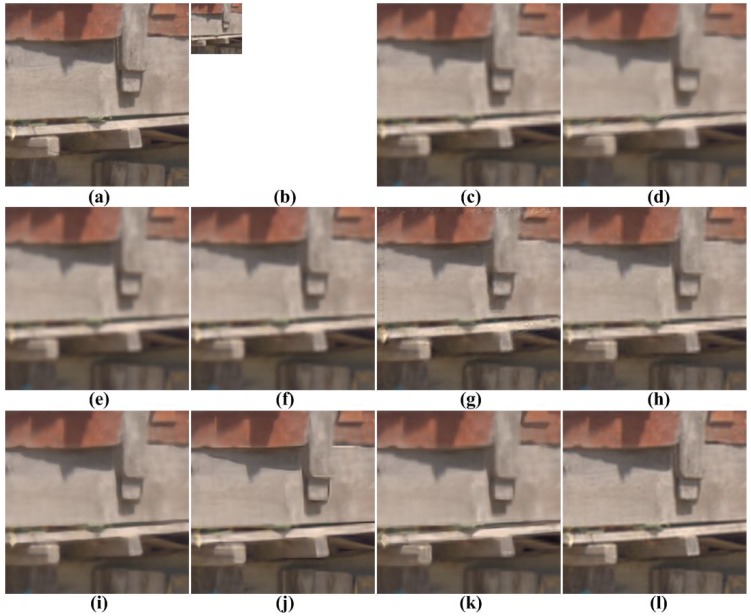
Results of resolution enhancement by enlarging a simulated LR multispectral image: (**a**) cropped original HR image in Figure 4e; (**b**) the four-times down-sampled LR image; results of: (**c**) cubic-spline interpolation [3], (**d**) interpolation-based SR [4], (**e**) interpolation-based SR [5], (**f**) interpolation-based SR [6], (**g**) example-based SR [7], (**h**) patch-based SR [9], (**i**) patch-based SR [10], (j) patch-based SR [11], (**k**) patch-based SR [12] and (**l**) the proposed method.

Figures 5, 6, 7, 8 and 9 show the results of enhancing the resolution of multispectral images using nine existing SR methods and the proposed multispectral SR method. Interpolation-based SR methods proposed in [3–6] commonly generate the blurring and jagging artifacts and cannot successfully recover the edge and texture details. The example-based method [7] and patch-based SR methods proposed in [9–12] can reconstruct clearer HR images than interpolation-based methods, but they cannot avoid unnatural artifacts in the neighborhood of the edge.

On the other hand, the proposed method shows a significantly improved SR result by successfully reconstructing the original high-frequency details and sharpens edges without unnatural artifacts. The PSNR, SSIM, MS-SSIM, FSIM, BRISQUE and NIQE values of the simulated multispectral test images shown in Figure 4 are computed for nine different methods, as summarized in Table 1.

Based on Table 1, the proposed method gives better results than existing SR methods in the sense of PSNR, SSIM, MS-SSIM and FSIM. Although the proposed method did not always provide the best results in the sense of NIQR and BRISQUE, the averaged performance using the extended set of test images shows that the proposed SR method performs the best.

**Figure 10:**
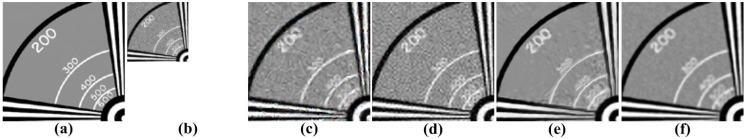
Results of resolution enhancement by enlarging a simulated noisy LR monochromatic image: (**a**) original HR image; (**b**) the two-times down-sampled LR image with additive white Gaussian noise (*σ* = 10); results of: (**c**) example-based SR [7], (**d**) patch-based SR [9]; (**e**) patch-based SR [12] and (**g**) the proposed method.

In the additional experiment, an original monochromatic HR image is down-sampled and added by zero-mean white Gaussian noise with standard deviation *σ* = 10 to obtain a simulated version of the noisy LR image. The simulated LR image is enlarged by three existing SR [7,9,12] and the proposed methods, as shown in Figure 10. As shown in Figure 10, existing SR methods can neither remove the noise, nor recover the details in the image, whereas the proposed method can successfully reduce the noise and successfully reconstruct the original details. Table 2 shows PSNR and SSIM values of three existing SR methods for the same test image shown in Figure 10.

The original version of the example-based SR method was not designed for real-time processing, since it requires a patch dictionary before starting the SR process [7]. The performance and processing time of the patch searching process also depend on the size of the dictionary. The sparse representation-based SR method needs iterative optimization for the ***ℓ*_1_** minimization process [9], which results in indefinite processing time. Although the proposed SR method also needs iterative optimization for the directionally-adaptive regularization, the regularized optimization can be replaced by an approximated finite-support spatial filter at the cost of the quality of the resulting images [31]. The non-local means filtering is another time-consuming process in the proposed work. However, a finite processing time can be guaranteed by restricting the search range of patches.

### 4.2. Experiment Using Real UAV Images

**Figure 11:**
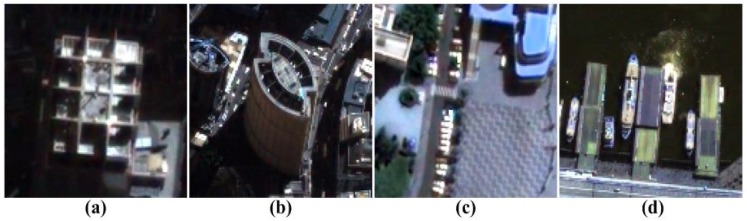
Four real UAV test images.

The proposed method is tested to enhance real UAV images, as shown Figure 11. More specifically, the remote sensing image is acquired by QuickBird equipped with a push broom-type image sensor to obtain a 0.65-m ground sample distance (GSD) panchromatic image.

**Figure 12:**
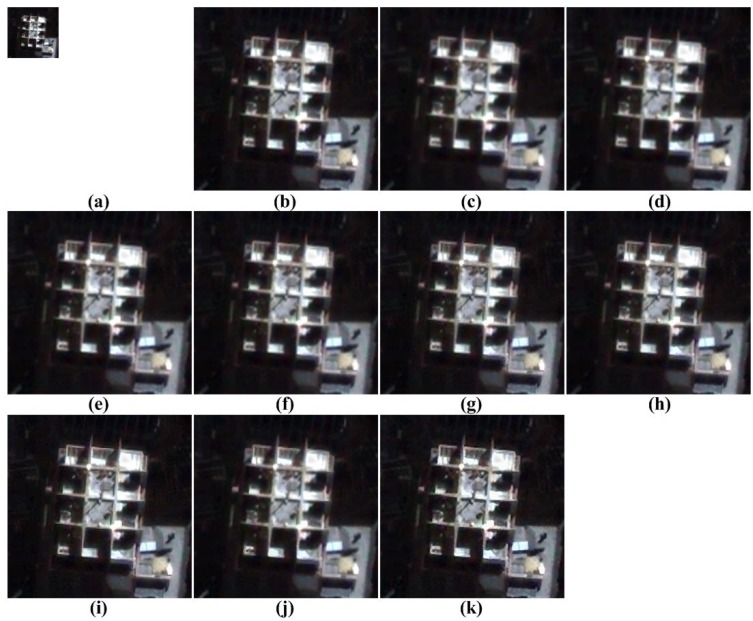
Results of resolution enhancement by enlarging a real UAV image: (**a**) original HR image in Figure 11a; result of: (**b**) cubic-spline interpolation [3]; (**c**) interpolation-based SR [4]; (**d**) interpolation-based SR [5]; (**e**) interpolation-based SR [6]; (**f**) example-based SR [7]; (**g**) patch-based SR [9]; (**h**) patch-based SR [10]; (**i**) patch-based SR [11]; (**j**) patch-based SR [12] and (**k**) the proposed method.

**Figure 13:**
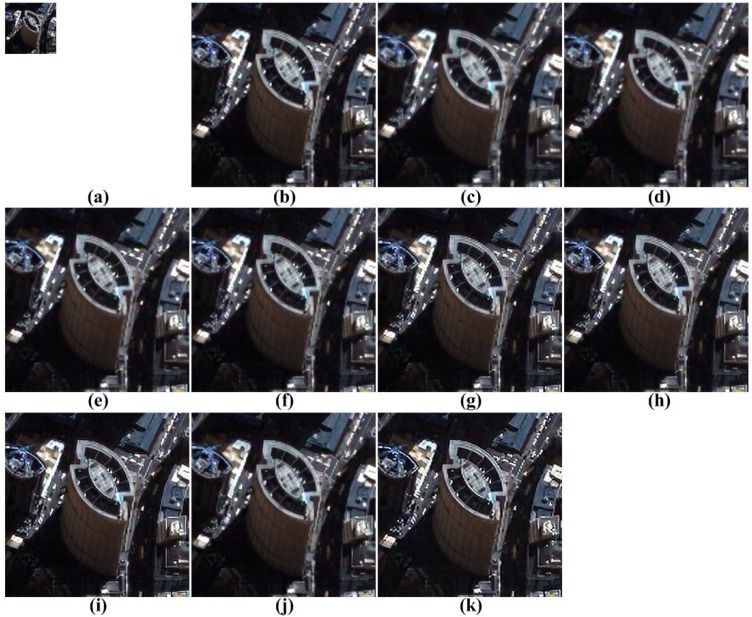
Results of resolution enhancement by enlarging a real UAV image: (**a**) original HR image in Figure 11b; result of: (**b**) cubic-spline interpolation [3]; (**c**) interpolation-based SR [4]; (**d**) interpolation-based SR [5]; (**e**) interpolation-based SR [6]; (**f**) example-based SR [7]; (**g**) patch-based SR [9]; (**h**) patch-based SR [10]; (**i**) patch-based SR [11]; (**j**) patch-based SR [12] and (**k**) the proposed method.

**Figure 14:**
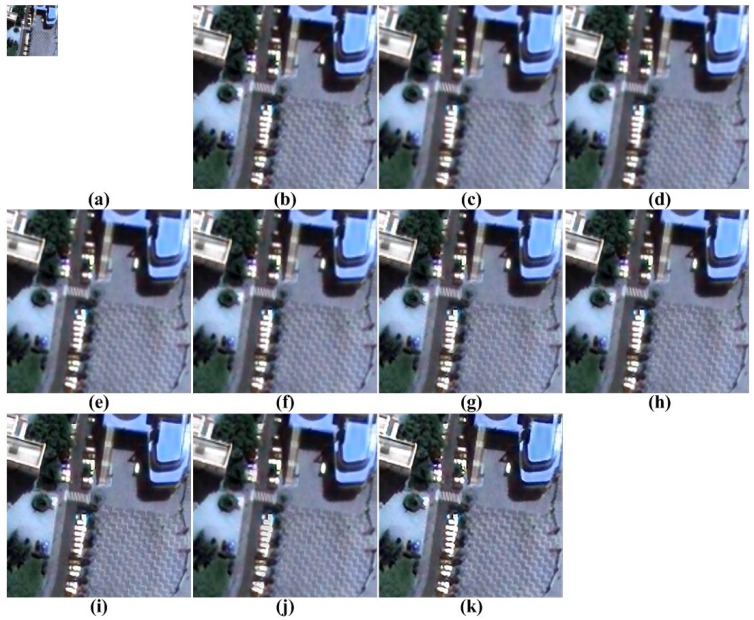
Results of resolution enhancement by enlarging a real UAV image: (**a**) original HR image in Figure 11c; result of: (**b**) cubic-spline interpolation [3]; (**c**) interpolation-based SR [4]; (**d**) interpolation-based SR [5]; (**e**) interpolation-based SR [6]; (**f**) example-based SR [7]; (**g**) patch-based SR [9]; (**h**) patch-based SR [10]; (**i**) patch-based SR [11]; (**j**) patch-based SR [12] and (**k**) the proposed method.

**Figure 15:**
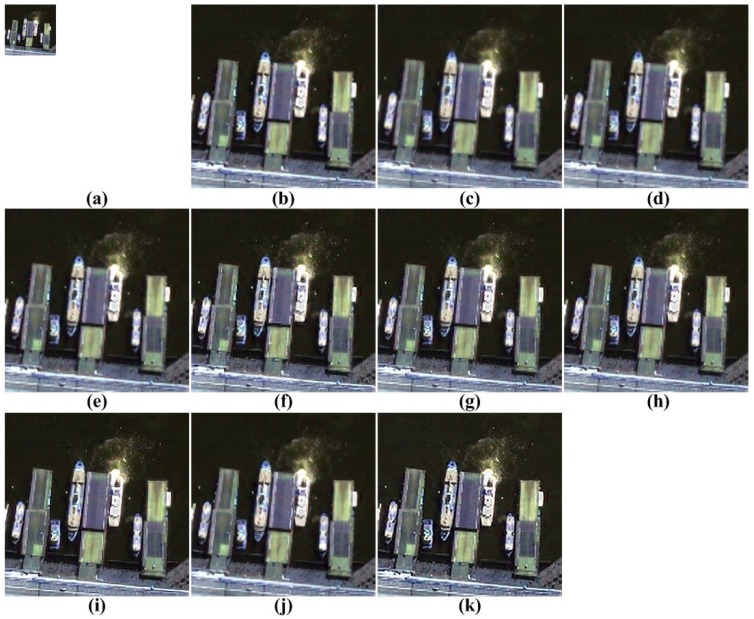
Results of resolution enhancement by enlarging a real UAV image: (**a**) original HR image in Figure 11d; result of: (**b**) cubic-spline interpolation [3]; (**c**) interpolation-based SR [4]; (**d**) interpolation-based SR [5]; (**e**) interpolation-based SR [6]; (**f**) example-based SR [7]; (**g**) patch-based SR [9]; (**h**) patch-based SR [10]; (**i**) patch-based SR [11]; (**j**) patch-based SR [12] and (**k**) the proposed method.

Figures 12, 13, 14 to 15 show the results of enhanced versions using nine different SR and the proposed methods. In order to obtain no-reference measures, such as NIQE and BRISQUE values, Figure 11a–d are four-times magnified. In addition, the original UAV images are four-times down-sampled to generate simulated LR images and compared by the full-reference image quality measures, as summarized in Table 3.

As shown in Figures 12, 13, 14 and 15, the interpolation-based SR methods cannot successfully recover the details in the image. Since they are not sufficiently close to the unknown HR image, their NIQE and BRISQUE values are high, whereas example-based SR methods generate unnatural artifacts near the edge because of the inappropriate training dataset. Patch-based and the proposed SR methods provide better SR results.

The PSNR, SSIM, MS-SSIM, FSIM, NIQE and BRISQUE values are computed using nine different SR methods, as summarized in Table 3. Based on Table 3, the proposed method gives better results than existing SR methods in the sense of PSNR, SSIM, MS-SSIM and FSIM. Although the proposed method did not always provide the best results in the sense of NIQE and BRISQUE, the averaged performance using the extended set of test images shows that the proposed SR method performs the best.

## 5. Conclusions

In this paper, we presented a multisensor super-resolution (SR) method using directionally-adaptive regularization and multispectral image fusion. The proposed method can overcome the physical limitation of a multispectral image sensor by estimating the color HR image from a set of multispectral LR images. More specifically, the proposed method combines the directionally-adaptive regularized image reconstruction and a modified multiscale non-local means (NLM) filter. As a result, the proposed SR method can restore the detail near the edge regions without noise amplification or unnatural SR artifacts. Experimental results show that the proposed method provided a better SR result than existing state-of-the-art methods in the sense of objective measures. The proposed method can be applied to all types of images, including a gray-scale or single-image, RGB color and multispectral images.

## Figures and Tables

**Table 1:** Comparison of peak-to-peak signal-to-noise ratio (PSNR), structural similarity index measure (SSIM), multiscale-SSIM (MS-SSIM), feature similarity index (FSIM), blind/referenceless image spatial quality evaluator (BRISQUE) and natural image quality evaluator (NIQE) values of the resulting images shown in Figure 4 using nine existing and the proposed SR methods.

**Images**	**Methods**	[3]	[4]	[5]	[6]	[7]	[9]	[10]	[11]	[12]	**Proposed**
Figure 4a	PSNR	28.13	24.82	25.03	28.50	25.84	30.14	30.21	-	27.49	**36.64**
	SSIM [26]	0.844	0.751	0.763	0.844	0.784	0.878	0.877	-	0.827	**0.964**
	MS-SSIM [27]	0.945	0.885	0.891	0.955	0.920	0.974	0.971	-	0.930	**0.993**
	FSIM [28]	0.875	0.825	0.830	0.877	0.854	0.903	0.905	-	0.863	**0.969**
	BRISQUE [29]	65.30	64.29	72.66	63.22	51.98	55.51	53.84	57.78	58.68	**48.30**
	NIQE [30]	9.65	11.18	12.45	8.47	7.50	9.07	11.35	9.29	8.46	**7.10**

Figure 4b	PSNR	27.76	24.94	25.09	26.12	25.21	28.97	29.28	-	27.27	**34.37**
	SSIM [26]	0.794	0.695	0.707	0.743	0.738	0.816	0.818	-	0.777	**0.931**
	MS-SSIM [27]	0.939	0.870	0.877	0.909	0.919	0.961	0.962	-	0.935	**0.990**
	FSIM [28]	0.855	0.795	0.804	0.823	0.843	0.869	0.871	-	0.848	**0.951**
	BRISQUE [29]	65.83	70.40	69.35	58.66	48.66	62.82	53.32	53.97	56.96	**48.33**
	NIQE [30]	9.65	16.13	11.37	7.97	7.15	9.91	8.47	8.12	8.41	**7.05**

Figure 4c	PSNR	25.04	24.69	24.91	25.03	26.54	28.68	25.51	-	26.29	**33.21**
	SSIM [26]	0.712	0.680	0.694	0.708	0.766	0.824	0.819	-	0.772	**0.932**
	MS-SSIM [27]	0.882	0.853	0.866	0.876	0.926	0.965	0.962	-	0.914	**0.987**
	FSIM [28]	0.817	0.782	0.793	0.810	0.850	0.870	0.858	-	0.834	**0.948**
	BRISQUE [29]	58.51	66.21	70.53	61.71	51.29	51.78	49.35	51.19	57.65	**49.06**
	NIQE [30]	8.59	13.35	13.00	8.12	7.07	**6.61**	7.15	7.17	7.91	6.79

Figure 4d	PSNR	23.22	23.08	23.22	23.24	24.77	29.73	29.86	-	27.34	**32.38**
	SSIM [26]	0.679	0.649	0.665	0.677	0.736	0.843	0.837	-	0.796	**0.939**
	MS-SSIM [27]	0.874	0.858	0.865	0.871	0.917	0.974	0.971	-	0.950	**0.988**
	FSIM [28]	0.824	0.793	0.804	0.818	0.853	0.895	0.886	-	0.867	**0.959**
	BRISQUE [29]	64.41	68.59	74.00	67.63	61.84	**56.83**	57.22	60.81	64.12	60.83
	NIQE [30]	8.17	13.85	11.49	8.22	7.12	7.14	7.43	**7.07**	8.32	7.85

Figure 4e	PSNR	26.43	26.45	26.37	26.49	26.80	32.45	33.25	-	29.26	**37.50**
	SSIM [26]	0.852	0.846	0.850	0.853	0.861	0.925	0.930	-	0.896	**0.969**
	MS-SSIM [27]	0.918	0.915	0.915	0.918	0.935	0.985	0.985	-	0.967	**0.996**
	FSIM [28]	0.872	0.856	0.869	0.873	0.883	0.927	0.930	-	0.895	**0.967**
	BRISQUE [29]	68.90	74.64	74.39	66.52	**51.99**	56.45	57.68	61.63	61.92	56.55
	NIQE [30]	9.93	13.78	10.64	9.91	9.86	**7.72**	9.07	8.97	8.94	8.37

Average	PSNR	26.12	24.79	24.93	25.88	25.83	29.99	29.62	-	27.53	**34.82**
	SSIM [26]	0.776	0.724	0.736	0.765	0.777	0.857	0.856	-	0.814	**0.947**
	MS-SSIM [27]	0.912	0.876	0.883	0.906	0.923	0.972	0.970	-	0.939	**0.991**
	FSIM [28]	0.849	0.810	0.820	0.840	0.857	0.893	0.890	-	0.861	**0.959**
	BRISQUE [29]	64.59	68.83	72.19	63.55	53.15	56.68	54.28	57.08	59.87	**52.61**
	NIQE [30]	9.20	13.66	11.79	8.54	7.74	8.09	8.69	8.12	8.41	**7.43**

**Table 2:** Comparison of PSNR and SSIM values of the resulting images using three existing SR and the proposed SR methods.

**Methods**	[7]	[9]	[12]	**Proposed**
PSNR	20.15	21.83	18.95	**24.45**
SSIM	0.566	0.523	0.802	**0.869**

**Table 3:** Comparison of PSNR, SSIM, MS-SSIM, FSIM, BRISQUE and NIQE values of the resulting images shown in Figure 11 using nine existing and the proposed SR methods.

**Images**	**Methods**	[3]	[4]	[5]	[6]	[7]	[9]	[10]	[11]	[12]	**Proposed**
Figure 11a	PSNR	17.85	17.56	17.75	17.82	18.61	21.94	21.97	-	19.12	**26.86**
	SSIM [26]	0.710	0.681	0.698	0.709	0.723	0.824	0.830	-	0.766	**0.930**
	MS-SSIM [27]	0.837	0.844	0.843	0.840	0.882	0.948	0.955	-	0.907	**0.990**
	FSIM [28]	0.795	0.784	0.791	0.795	0.812	0.855	0.589	-	0.823	**0.929**
	BRISQUE [29]	63.35	38.87	71.87	63.29	63.91	52.78	55.73	58.04	64.69	**50.90**
	NIQE [30]	8.31	10.24	10.10	8.50	8.92	7.30	7.83	7.24	8.51	**5.97**

Figure 11b	PSNR	18.14	18.82	18.86	18.21	18.89	19.60	19.82	-	18.19	**22.53**
	SSIM [26]	0.641	0.654	0.662	0.666	0.618	0.670	0.680	-	0.590	**0.824**
	MS-SSIM [27]	0.821	0.840	0.825	0.825	0.886	0.915	0.922	-	0.853	**0.971**
	FSIM [28]	0.772	0.778	0.780	0.790	0.740	0.769	0.768	-	0.721	**0.865**
	BRISQUE [29]	52.93	46.00	67.31	50.33	46.24	51.71	39.59	41.50	39.03	**37.12**
	NIQE [30]	6.81	8.08	9.80	6.21	6.63	6.53	6.77	4.76	**4.71**	5.36

Figure 11c	PSNR	19.89	19.81	20.29	19.98	20.62	21.64	21.74	-	20.14	**24.94**
	SSIM [26]	0.634	0.609	0.651	0.643	0.615	0.660	0.661	-	0.600	**0.856**
	MS-SSIM [27]	0.805	0.802	0.822	0.807	0.876	0.903	0.897	-	0.847	**0.973**
	FSIM [28]	0.815	0.796	0.813	0.826	0.780	0.812	0.811	-	0.776	**0.901**
	BRISQUE [29]	61.30	63.46	71.18	63.32	64.93	53.94	**53.28**	54.58	62.75	55.88
	NIQE [30]	7.75	9.83	10.10	7.94	8.66	6.58	7.18	6.63	7.63	**5.85**

Figure 11d	PSNR	18.68	19.00	19.07	18.52	19.59	20.36	20.47	-	18.99	**23.98**
	SSIM [26]	0.633	0.646	0.656	0.646	0.635	0.679	0.686	-	0.635	**0.841**
	MS-SSIM [27]	0.818	0.828	0.835	0.816	0.882	0.912	0.910	-	0.872	**0.973**
	FSIM [28]	0.776	0.776	0.780	0.778	0.746	0.778	0.776	-	0.753	**0.873**
	BRISQUE [29]	55.20	52.13	68.70	54.06	51.35	53.70	**44.93**	50.01	48.33	47.51
	NIQE [30]	7.11	8.69	9.48	6.66	5.06	5.65	5.96	5.11	5.33	**5.56**

Average	PSNR	18.64	18.80	18.99	18.63	19.43	20.88	21.00	-	19.11	**24.58**
	SSIM [26]	0.654	0.648	0.667	0.666	0.648	0.708	0.714	-	0.648	**0.863**
	MS-SSIM [27]	0.820	0.828	0.831	0.822	0.881	0.919	0.921	-	0.870	**0.977**
	FSIM [28]	0.789	0.783	0.791	0.797	0.770	0.803	0.736	-	0.768	**0.892**
	BRISQUE [29]	58.19	50.12	69.77	57.75	56.61	53.03	48.38	51.03	53.70	**47.85**
	NIQE [30]	7.49	9.21	9.87	7.33	7.32	6.52	6.94	5.93	6.55	**5.68**
